# Supplementary material for: Targeting endothelin 1 receptor-miR-200b/c-ZEB1 circuitry blunts metastatic progression in ovarian cancer
Source: Commun Biol. 2020 Nov 13;3:677. doi: 10.1038/s42003-020-01404-3 (PMC7666224; doi:10.1038/s42003-020-01404-3)
Supplement: Supplementary file 1 — Supplementary Information [file 42003_2020_1404_MOESM1_ESM.pdf]

Supplementary Information

Targeting endothelin 1 receptor-miR-200b/c-ZEB1 circuitry blunts metastatic progression in ovarian cancer

Rosanna Sestito, Roberta Cianfrocca, Piera Tocci, Laura Rosanò, Andrea Sacconi, Giovanni Blandino and Anna Bagnato

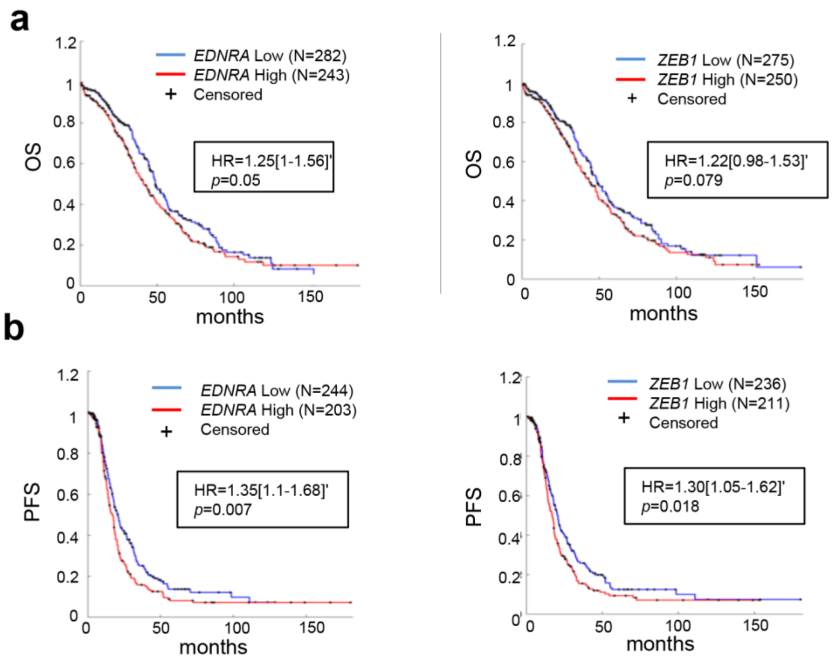

Supplementary Figure 1. High levels of ET$_A$R or ZEB1 correlate with a poor prognosis in ovarian cancer patients. a Kaplan-Meier curves of overall survival (OS) of 525 patients from TCGA subdivided in high (z score > 1.5, red line) and low (z score < 1.5, blue line) expression levels of *EDNRA* (ET$_A$R) or *ZEB1* genes. **b** Kaplan-Meier curves of progression free survival (PFS) of 447 patients from TCGA subdivided as in **a**.

a

ETAR miRanda	ETAR Targetscan	ETAR miRmap
hsa-miR-1	hsa-miR-1271	hsa-miR-128
hsa-miR-1271	hsa-miR-128	hsa-miR-181b-3p
hsa-miR-132	hsa-miR-128-3p	**hsa-miR-200b-3p**
hsa-miR-135a	hsa-miR-132-3p	**hsa-miR-200c-3p**
hsa-miR-135b	hsa-miR-135-5p	hsa-miR-216a-5p
hsa-miR-137	hsa-miR-135a	hsa-miR-27a-3p
hsa-miR-148a	hsa-miR-135b	hsa-miR-27b-3p
hsa-miR-148b	hsa-miR-137	hsa-miR-2861
hsa-miR-149	hsa-miR-140-3p.1	hsa-miR-3140-5p
hsa-miR-152	hsa-miR-140-3p.2	hsa-miR-3143
hsa-miR-190	hsa-miR-148-3p	hsa-miR-335-3p
hsa-miR-190b	hsa-miR-152-3p	hsa-miR-3607-3p
hsa-miR-197	hsa-miR-15-5p	hsa-miR-3681-3p
**hsa-miR-200b**	hsa-miR-16-5p	hsa-miR-3686
**hsa-miR-200c**	hsa-miR-181-5p	hsa-miR-376a-5p
hsa-miR-203	hsa-miR-182-5p	hsa-miR-3918
hsa-miR-212	hsa-miR-183-5p.2	hsa-miR-4252
hsa-miR-216b	hsa-miR-190-5p	hsa-miR-4265
hsa-miR-217	hsa-miR-193-3p	**hsa-miR-429**
hsa-miR-218	hsa-miR-194-5p	hsa-miR-4296
hsa-miR-224	hsa-miR-195-5p	hsa-miR-4312
hsa-miR-27a	**hsa-miR-200b-3p**	hsa-miR-4322
hsa-miR-27b	**hsa-miR-200c-3p**	hsa-miR-4420
hsa-miR-299-3p	hsa-miR-202-5p	hsa-miR-4422
hsa-miR-30a	hsa-miR-203	hsa-miR-4512
hsa-miR-30b	hsa-miR-212-3p	hsa-miR-4769-3p
hsa-miR-30c	hsa-miR-212-5p	hsa-miR-5011-5p
hsa-miR-30d	hsa-miR-216-3p	hsa-miR-622
hsa-miR-30e	hsa-miR-216a-5p	hsa-miR-6724-5p
hsa-miR-329	hsa-miR-216b-5p	hsa-miR-766-5p
hsa-miR-361-5p	hsa-miR-217	
hsa-miR-362-3p	hsa-miR-218-5p	
hsa-miR-365	hsa-miR-221-3p	
hsa-miR-374a	hsa-miR-222-3p	
hsa-miR-374b	hsa-miR-223-3p	
hsa-miR-377	hsa-miR-24-3p	
hsa-miR-383	hsa-miR-27-3p	
hsa-miR-425	hsa-miR-27a	
**hsa-miR-429**	hsa-miR-27b	
hsa-miR-433	hsa-miR-302-3p.2	
hsa-miR-448	hsa-miR-3064-5p	
hsa-miR-490-3p	hsa-miR-30a	
hsa-miR-543	hsa-miR-30b	
hsa-miR-599	hsa-miR-30c	
hsa-miR-653	hsa-miR-30d	
hsa-miR-96	hsa-miR-30e	
	hsa-miR-338-3p	
	hsa-miR-365-3p	
	hsa-miR-3681-3p	
	hsa-miR-383-5p.1	
	hsa-miR-383-5p.2	
	hsa-miR-424-5p	
	hsa-miR-425-5p	
	hsa-miR-4262	
	**hsa-miR-429**	
	hsa-miR-455-3p.1	
	hsa-miR-489-3p	
	hsa-miR-490-3p	
	hsa-miR-497-5p	
	hsa-miR-520-3p	
	hsa-miR-6504-5p	
	hsa-miR-6807-3p	
	hsa-miR-6838-5p	
	hsa-miR-96	

ZEB1 miRanda	ZEB1 Targetscan	ZEB1 miRmap
hsa-miR-101	hsa-miR-217	hsa-miR-4717-5p
hsa-miR-1271	hsa-miR-101-3p.1	hsa-miR-105-5p
hsa-miR-128	hsa-miR-101-3p.1	hsa-miR-1202
hsa-miR-130a	hsa-miR-105-5p	hsa-miR-1208
hsa-miR-130b	hsa-miR-10-5p	hsa-miR-128
hsa-miR-136	hsa-miR-124-3p,2	hsa-miR-141
hsa-miR-139-5p	hsa-miR-124-3p.1	hsa-miR-200a-3p
hsa-miR-140-5p	hsa-miR-1271-5p	**hsa-miR-200b-3p**
hsa-miR-141	hsa-miR-128-3p	**hsa-miR-200c-3p**
hsa-miR-142-3p	hsa-miR-130-3p	hsa-miR-208a
hsa-miR-143	hsa-miR-139-5p	hsa-miR-208b
hsa-miR-144	hsa-miR-1-3p	hsa-miR-216a-3p
hsa-miR-150	hsa-miR-140-3p.2	hsa-miR-301a-5p
hsa-miR-181a	hsa-miR-140-5p	hsa-miR-3154
hsa-miR-181b 1572	hsa-miR-141-3p	hsa-miR-3163
hsa-miR-181c	hsa-miR-142-3p.1	hsa-miR-340-5p
hsa-miR-181d	hsa-miR-142-3p.2	hsa-miR-34a-3p
hsa-miR-183	hsa-miR-143-3p	hsa-miR-3681-3p
hsa-miR-186	hsa-miR-144-3p	hsa-miR-369-3p
hsa-miR-196a	hsa-miR-146-5p	hsa-miR-374c-5p
hsa-miR-196b	hsa-miR-181-5p	hsa-miR-3972
hsa-miR-199a-5p	hsa-miR-183-5.1	hsa-miR-4269
hsa-miR-199b-5p	hsa-miR-183-5p,2	**hsa-miR-429**
hsa-miR-200a	hsa-miR-194-5p	hsa-miR-4755-3p
**hsa-miR-200b**	hsa-miR-196-5p	hsa-miR-4803
**hsa-miR-200c**	hsa-miR-199-3p	hsa-miR-499a-5p
hsa-miR-203	hsa-miR-199-5p	hsa-miR-5006-5p
hsa-miR-204	hsa-miR-200a-3p	hsa-miR-548a-3p
hsa-miR-205	**hsa-miR-200b-3p**	hsa-miR-548ar-3p
hsa-miR-208a	**hsa-miR-200c-3p**	hsa-miR-548av-3p
hsa-miR-208b	hsa-miR-203a-3p.1	hsa-miR-548az-3p
hsa-miR-211	hsa-miR-204-5p	hsa-miR-548e
hsa-miR-216a	hsa-miR-205-5p	hsa-miR-548f
hsa-miR-217	hsa-miR-206	hsa-miR-551b-5p
hsa-miR-219-5p	hsa-miR-211-5p	hsa-miR-5702
hsa-miR-223	hsa-miR-216a-5p	hsa-miR-629-3p
hsa-miR-23a	hsa-miR-217	hsa-miR-655
hsa-miR-23b	hsa-miR-219-5p	hsa-miR-6715b-5p
hsa-miR-300	hsa-miR-223-3p	hsa-miR-892a
hsa-miR-301a	hsa-miR-23-3p	
hsa-miR-301b	hsa-miR-27-3p	
hsa-miR-32 1	hsa-miR-301-3p	
hsa-miR-33a	hsa-miR-34-5p	
hsa-miR-33b	hsa-miR-383-5p.2	
hsa-miR-340	hsa-miR-425-5p	
hsa-miR-342-3p	**hsa-miR-429**	
hsa-miR-361-5p	hsa-miR-454-3p	
hsa-miR-375	hsa-miR-455-3p.1	
hsa-miR-377	hsa-miR-499-5p	
hsa-miR-381	hsa-miR-499a-5p	
hsa-miR-410	hsa-miR-506-3p	
**hsa-miR-429**	hsa-miR-96-5p	
hsa-miR-431		
hsa-miR-448		
hsa-miR-454		
hsa-miR-494		
hsa-miR-499-5p		
hsa-miR-539		
hsa-miR-590-3p		
hsa-miR-758		
hsa-miR-873		
hsa-miR-96		

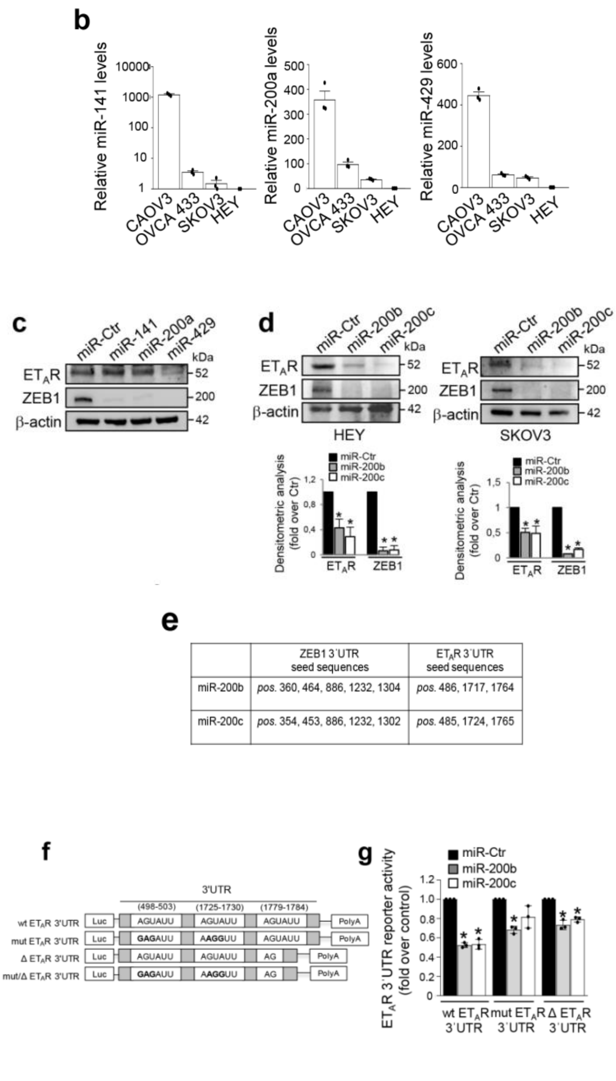

Supplementary Figure 2. ET$_A$R and ZEB1 are putative targets of miR-200b/c. a miRNAs predicted to target the 3'UTR of ET$_A$R or the 3'UTR of ZEB1 by using miRanda, TargetScan and miRmap bioinformatic tools. **b** miR-141, miR-200a and miR-429 expression levels in CAOV3, OVCA 433, SKOV3 and HEY cell lines analyzed by qRT-PCR and normalized to U6. **c** WB analysis of ET$_A$R and ZEB1 protein expression in HEY cells transfected with mimic-miRNA Ctr (miR-Ctr), mimic-miR-141 (miR-141) or mimic-miR-200a (miR-200a) or mimic-miR-429 (miR-429) for 48h. β-actin is used as loading control. **d** WB analysis of ET$_A$R and ZEB1 protein expression in HEY and SKOV3 cells transfected with mimic-miR-Ctr, -miR-200b, or -miR-200c for 48h. β-actin is used as loading control. Graph represents the densitometric analysis of ET$_A$R and

ZEB1 protein expression normalized to β-actin. Values are the mean ±SD expressed as fold induction (*, *p*<0.04 vs miR-Ctr). **e** miR-200b/c seed binding sequences on the 3'UTR of ZEB1 and ET_AR. **f** Schematic representation of the ET_AR 3'UTR reporter plasmids employed in the luciferase assays: wt ET_AR 3'UTR, mut ET_AR 3'UTR (carrying mutations in pos. 498-500 and 1726-1728), ΔET_AR 3'UTR (carrying a deletion of the region 1781-1784) and mut/ΔET_AR 3'UTR (carrying the mutations in pos. 498-500 and 1726-1728 and a deletion of the region 1781-1784). **g** Luciferase activity in HEY cells co-transfected with mimic-miR-Ctr, -miR-200b or -miR-200c together with the wt ET_AR 3'UTR, mut ET_AR 3'UTR or ΔET_AR 3'UTR reporter plasmids. Values are the mean ±SD expressed as fold induction (n=3; *, *p*<0.001 vs Ctr).

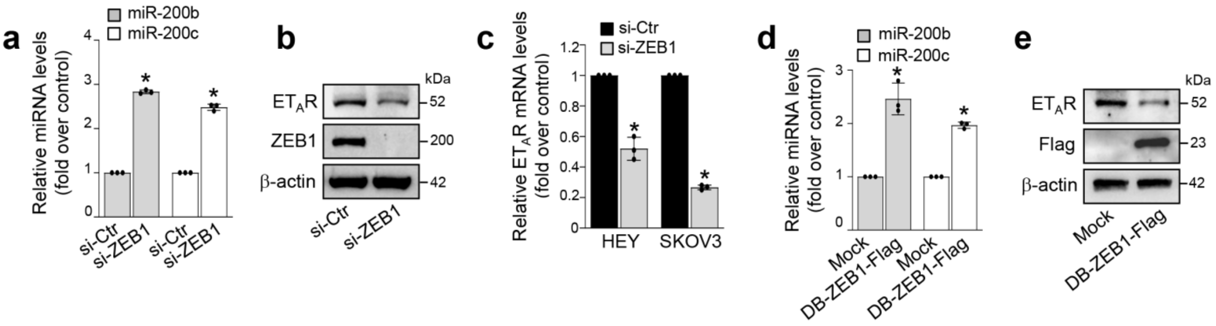

Supplementary Figure 3. ZEB1 regulates ET_AR expression through the suppression of miR-200b/c. **a** Expression of miR-200b/c in SKOV3 cells transfected for 72h with siRNA control (si-Ctr) or si-ZEB1 analyzed by qRT-PCR. U6 is used to normalize. Values are the mean ±SD (n=3; *, *p*<0.0001 vs si-Ctr). **b** Expression of ET_AR and ZEB1 proteins in SKOV3 cells transfected as in **a** and analyzed by WB. β-actin is used as loading control. **c** Expression levels of ET_AR mRNA in HEY and SKOV3 cells transfected as in **a** evaluated by qRT-PCR and normalized to cyclophilin-A. Values are the mean ±SD (n=3; *, *p*<0.001 vs si-Ctr). **d** qRT-PCR analyses to detect miR-200b/c expression in HEY cells transfected with a control plasmid (Mock) or with a Flag-tagged construct able to recognize and block the DNA binding domain of ZEB1 (DB-ZEB1-Flag) for 48h. U6 is used to normalize miRNA expression. Values are the mean ±SD (n=3; *, *p*<0.01 vs Mock). **e**

Expression of ET_AR and Flag proteins in HEY cells transfected as in **d** and analyzed by WB. β-actin is used as loading control.

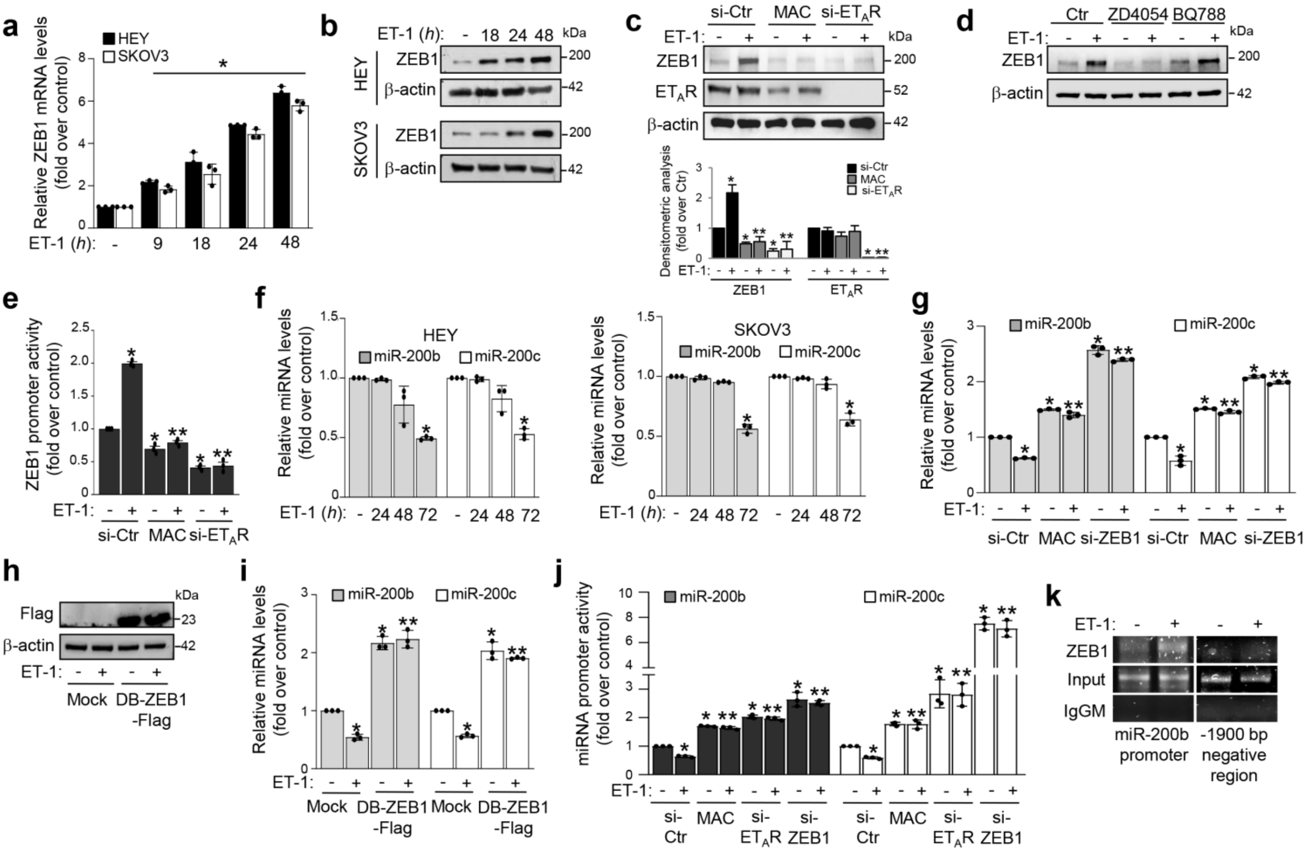

Supplementary Figure 4. ET-1 downregulates miR-200b/c via ZEB1. a, b ZEB1 mRNA (**a**) and protein (**b**) levels evaluated by qRT-PCR and WB and normalized to cyclophilin-A and β-actin in ET-1-stimulated HEY and SKOV3 cells for different times. Values are the mean ±SD (n=3; *, p<0.01 vs unstimulated cells). **c** Expression of ZEB1 and ET_AR in HEY cells transfected with si-Ctr, or si-ET_AR or treated with MAC and stimulated or not with ET-1 for 48h. β-actin is used as loading control. Graph represents the quantification of ZEB1 and ET_AR expression normalized to β-actin. Value are the mean ±SD (*, p<0.05 vs si-Ctr; **, p<0.05 vs ET-1 stimulated si-Ctr)**. d** Expression of ZEB1 in HEY cells treated with the selective ET_AR antagonist ZD4054 (1µM) and the selective ET_BR antagonist BQ788 (1µM) and stimulated or not with ET-1. β-actin is used as

loading control. **e** Luciferase activity in SKOV3 cells co-transfected for 48h with ZEB1 promoter reporter plasmid and treated as in **c**. Values are the mean ±SD (n=3; *, $p<0.002$ vs si-Ctr; **, $p<0.001$ vs ET-1 stimulated si-Ctr). **f** qRT-PCR for miR-200b/c expression in HEY and SKOV3 cells stimulated or not with ET-1 for different times. U6 is used to normalize. Values are the mean ±SD (n=3; *, $p<0.001$ vs unstimulated cells). **g** qRT-PCR for miR-200b/c expression in SKOV3 cells transfected with si-Ctr or si-ZEB1 or treated with MAC and stimulated with ET-1 for 72h. U6 is used to normalize. Values are the mean ±SD (n=3; *, $p<0.0001$ vs si-Ctr; **, $p<0.0001$ vs ET-1-stimulated si-Ctr). **h, i** WB analysis (**h**) and qRT-PCR for miR-200b/c expression (**i**) in HEY cells transfected with a control plasmid (Mock) or with the DB-ZEB1-Flag plasmid for 48h and stimulated or not with ET-1 for 72h. β-actin and U6 are used to normalize. Values are the mean ±SD (n=3; *, $p<0.001$ vs Mock **, $p<0.0001$ vs ET-1-stimulated Mock). **j** Luciferase activity in SKOV3 cells co-transfected for 48h with the reporter plasmids for miR-200b/c promoters and si-Ctr, si-ET_AR or si-ZEB1 or treated with MAC and stimulated or not with ET-1 for 24h. Values are the mean ±SD (n=3; *, $p<0.003$ vs si-Ctr; **, $p<0.001$ vs ET-1-stimulated si-Ctr). **k** The binding of ZEB1 on miR-200b promoter region and on a region -1900 bp upstream the miR-200b TSS is analyzed in SKOV3 cells treated or not with ET-1 for 24h by ChIP assays followed by PCR. Anti-IgG mouse (IgGM) Ab is used as control for all ChIP reactions.

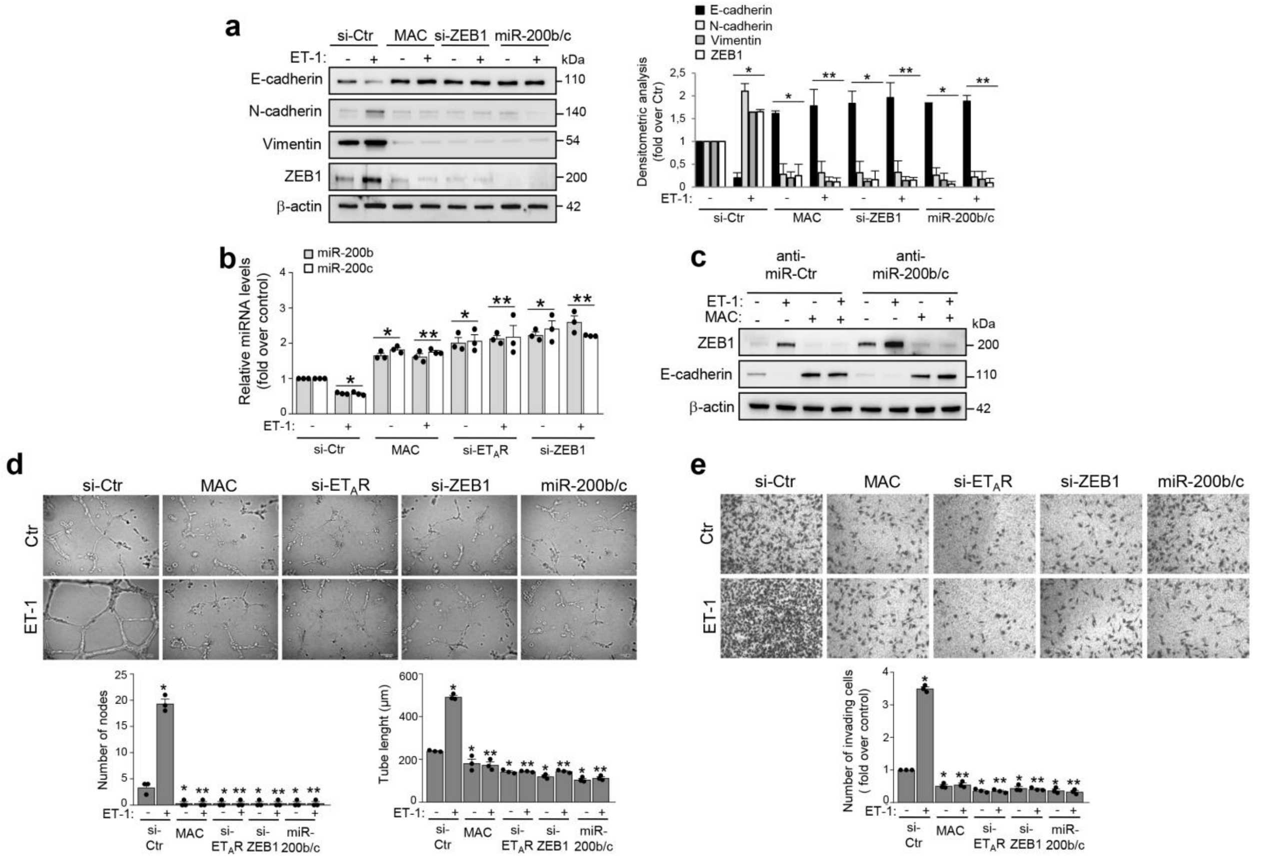

Supplementary Figure 5. The integrated ET$_A$R-miR-200b/c-ZEB1 circuit is involved in ET-1-dependent cell plasticity and invasion. **a** Expression of E-cadherin, N-cadherin, Vimentin and ZEB1 proteins is analyzed by WB in SKOV3 cells transfected with si-Ctr, si-ZEB1, or mimic-miR-200b/c or treated with MAC and stimulated or not with ET-1. β-actin is used as loading control. Graph represents the densitometric analysis of E-cadherin, N-cadherin, Vimentin and ZEB1 expression normalized to β-actin. Values are the mean ±SD expressed as fold induction (*, p<0.05 vs si-Ctr; **, p<0.05 vs ET-1 stimulated si-Ctr). **b** miR-200b/c expression in SKOV3 cells transfected with si-Ctr, si-ET$_A$R, si-ZEB1, or mimic-miR-200b/c and treated as in **a** is analyzed by qRT-PCR and normalized to U6. Values are the mean ±SD (n=3; *, p<0.004 vs si-Ctr; **, p<0.002 vs ET-1 stimulated si-Ctr). **c** Expression of ZEB1 and E-cadherin in SKOV3 cells transfected with anti-miR-Ctr or anti-miR-200b/c and treated or not with MAC and/or stimulated with ET-1. β-actin is used as loading control. **d** Assay of tubule-like structure formation in HEY cells transfected for 48h with siRNA control (si-Ctr), si-ET$_A$R, si-ZEB1, or mimic-miR-200b/c (miR-200b/c) or treated

with MAC and overnight stimulated or not with ET-1. Original magnification 20×. (Scale bar: 100μm). Graphs represent the number of nodes and the tube length in HEY cells. Columns show the mean ±SD (n=3; *, *p*<0.05 vs si-Ctr; **, *p*<0.0001 vs ET-1-stimulated si-Ctr). **e** Chemoinvasion assay in HEY cells transfected and stimulated as in **d**. Images represent the crystal violet-stained invasive cells. Magnification x10. Graph represents the number of invading HEY cells. Columns show the mean ±SD (n=3; *, *p*<0.001 vs si-Ctr; **, *p*<0.0001 vs ET-1-stimulated si-Ctr).

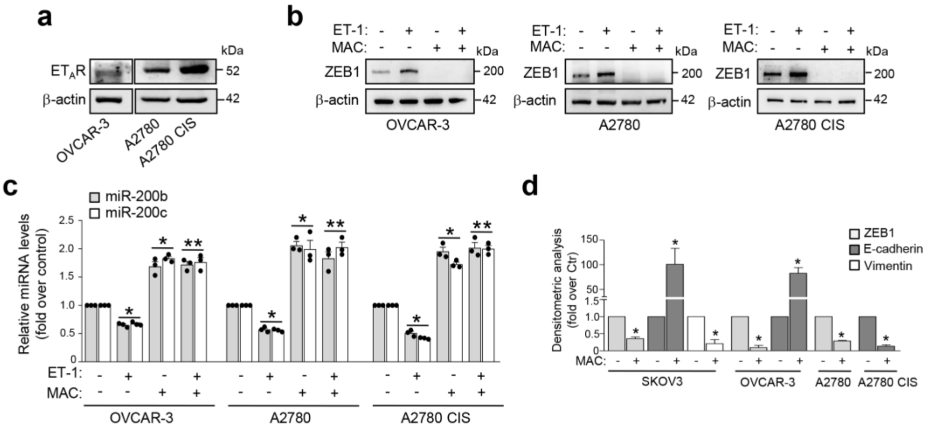

Supplementary Figure 6. Macitentan hampers ET-1/ZEB1-dependent miR-200b/c transcriptional regulation in high-grade serous and in chemoresistant ovarian cancer cell lines. a Expression of ET_AR in OVCAR-3, A2780 and A2780 CIS cells. β-actin is used as loading control. **b** Expression of ZEB1 in OVCAR-3, A2780 and A2780 CIS cell treated or not with MAC and/or stimulated with ET-1. β-actin is used as loading control. **c** qRT-PCR for miR-200b/c expression in OVCAR-3, A2780 and A2780 CIS cells treated or not with MAC and/or stimulated with ET-1. U6 is used to normalize. Values are the mean ±SD (n=3; *, *p*<0.02 vs unstimulated cells; **, *p*<0.02 vs ET-1-stimulated cells). **d** Densitometric analysis of ZEB1, E-cadherin, and Vimentin expression in SKOV3, OVCAR-3, A2780 and A2780 CIS xenografts normalized to β-actin. Values are the mean ±SD (*, *p*<0.04 vs Ctr).

Figure 1a

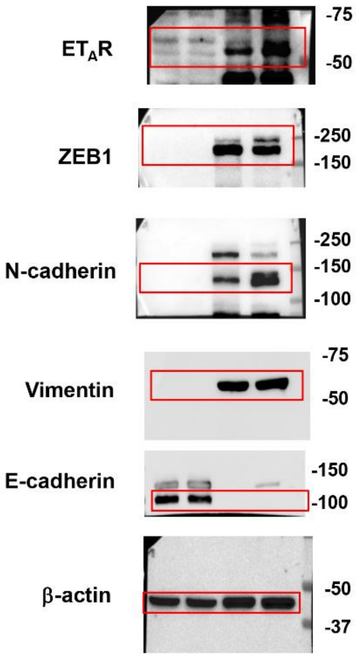

Figure 2d

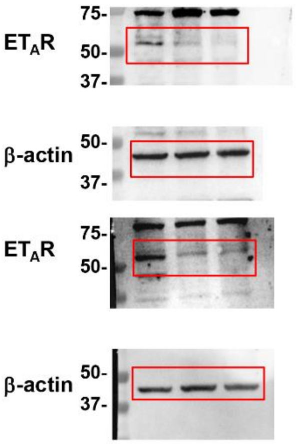

Figure 2f

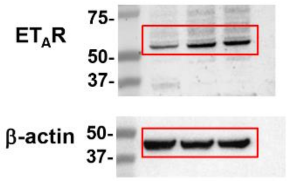

Figure 2h

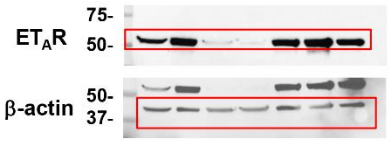

Figure 3c

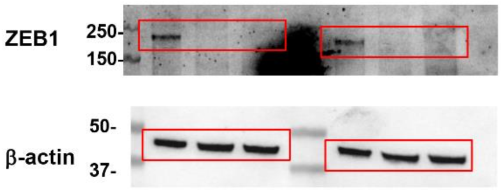

Figure 3e

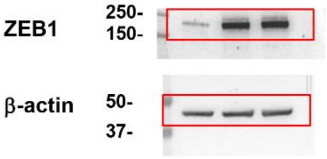

Figure 4b

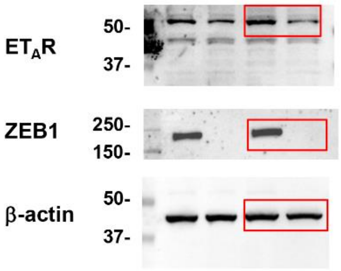

Figure 4e

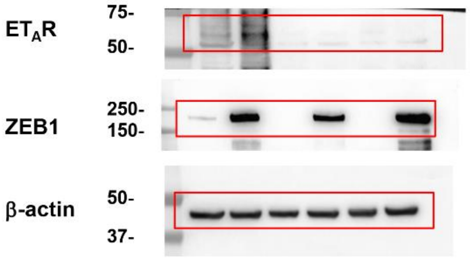

Figure 5a

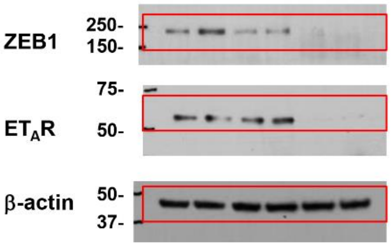

Figure 6a

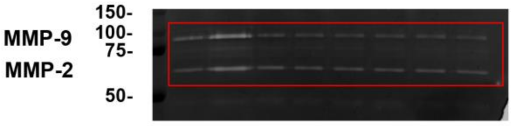

Figure 6b

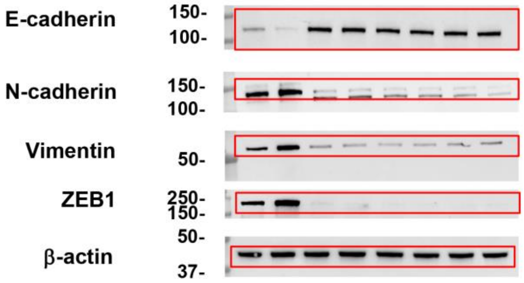

Figure 7c

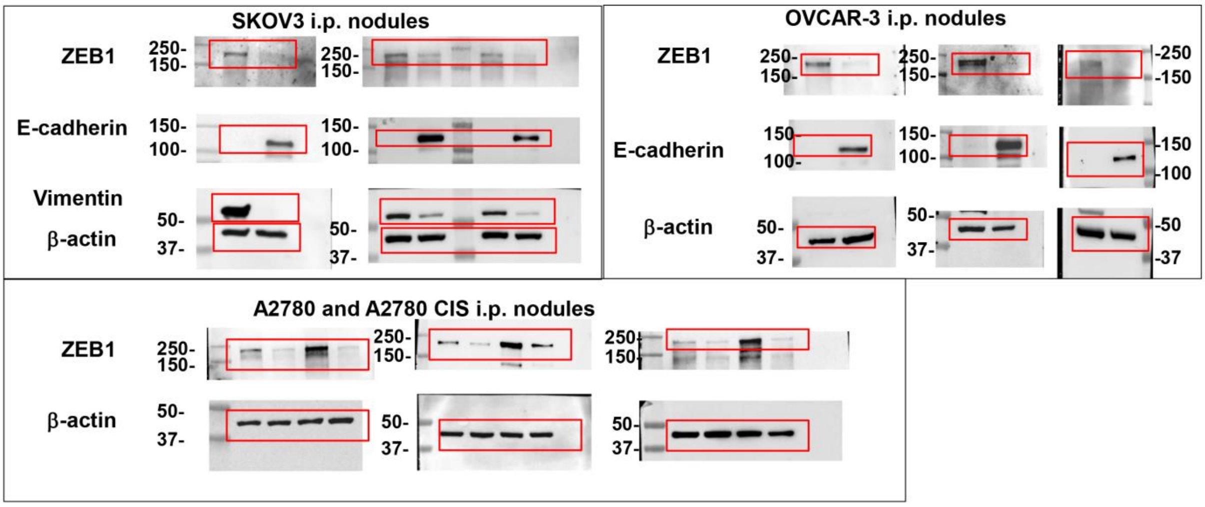

Figure 7f

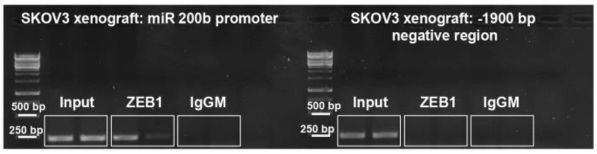

Supplementary Fig. 2c

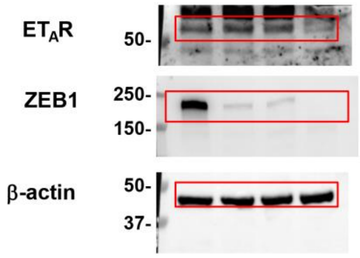

Supplementary Fig. 2d

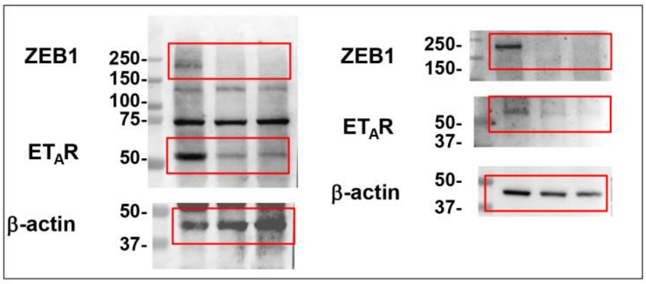

Supplementary Fig. 3b

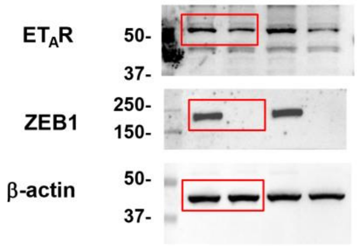

Supplementary Fig. 3e

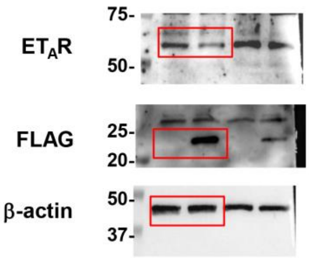

Supplementary Fig. 4b

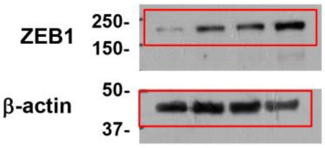

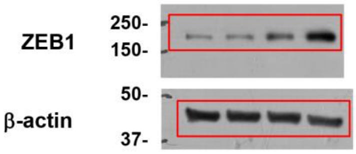

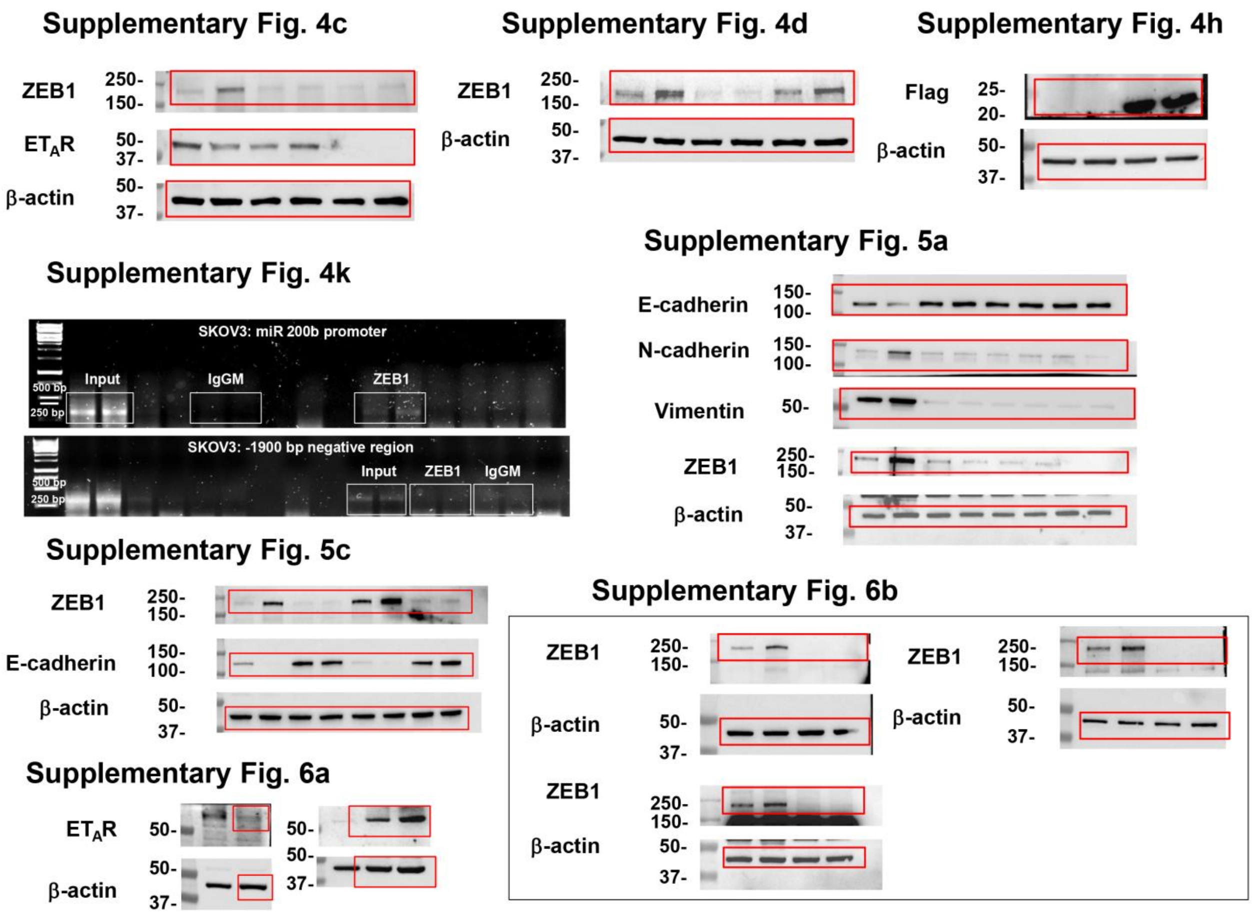

Supplementary Figure 7. Uncropped blots of figures 1-7 and supplementary figures 2-6.
