# Supplementary material for: Targeting endothelin 1 receptor-miR-200b/c-ZEB1 circuitry blunts metastatic progression in ovarian cancer
Source: Commun Biol. 2020 Nov 13;3:677. doi: 10.1038/s42003-020-01404-3 (PMC7666224; doi:10.1038/s42003-020-01404-3)
Supplement: Supplementary file 3 — Description of Additional Supplementary Files [file 42003_2020_1404_MOESM3_ESM.pdf]

Description of Additional Supplementary Files

File Name: Supplementary Data 1
Description: Source data underlying the graphs in the main figures
