# Supplementary material for: Targeting endothelin 1 receptor-miR-200b/c-ZEB1 circuitry blunts metastatic progression in ovarian cancer
Source: Commun Biol. 2020 Nov 13;3:677. doi: 10.1038/s42003-020-01404-3 (PMC7666224; doi:10.1038/s42003-020-01404-3)
Supplement: Supplementary file 5 — Reporting Summary [file 42003_2020_1404_MOESM5_ESM.pdf]

nature research

Corresponding author(s): Anna Bagnato

Last updated by author(s): Oct 9, 2020

Reporting Summary

Nature Research wishes to improve the reproducibility of the work that we publish. This form provides structure for consistency and transparency in reporting. For further information on Nature Research policies, see Authors & Referees and the Editorial Policy Checklist .

Statistics

For all statistical analyses, confirm that the following items are present in the figure legend, table legend, main text, or Methods section.

n/a	Confirmed	
☐	☒	The exact sample size (*n*) for each experimental group/condition, given as a discrete number and unit of measurement
☐	☒	A statement on whether measurements were taken from distinct samples or whether the same sample was measured repeatedly
☐	☒	The statistical test(s) used AND whether they are one- or two-sided<br>*Only common tests should be described solely by name; describe more complex techniques in the Methods section.*
☒	☐	A description of all covariates tested
☐	☒	A description of any assumptions or corrections, such as tests of normality and adjustment for multiple comparisons
☐	☒	A full description of the statistical parameters including central tendency (e.g. means) or other basic estimates (e.g. regression coefficient) AND variation (e.g. standard deviation) or associated estimates of uncertainty (e.g. confidence intervals)
☒	☐	For null hypothesis testing, the test statistic (e.g. *F*, *t*, *r*) with confidence intervals, effect sizes, degrees of freedom and *P* value noted<br>*Give P values as exact values whenever suitable.*
☒	☐	For Bayesian analysis, information on the choice of priors and Markov chain Monte Carlo settings
☒	☐	For hierarchical and complex designs, identification of the appropriate level for tests and full reporting of outcomes
☒	☐	Estimates of effect sizes (e.g. Cohen's *d*, Pearson's *r*), indicating how they were calculated

Our web collection on statistics for biologists contains articles on many of the points above.

Software and code

Policy information about availability of computer code

Data collection	No software code was used for data collection.
Data analysis	MATLAB software was used for the analysis of TCGA database; vasculogenic mimicry was analyzed using the Angiogenesis analyzer for ImageJ; grey values for Western Blotting were analyzed using ImageJ; statistical analyses were performed with Graphpad Prism 8

For manuscripts utilizing custom algorithms or software that are central to the research but not yet described in published literature, software must be made available to editors/reviewers. We strongly encourage code deposition in a community repository (e.g. GitHub). See the Nature Research guidelines for submitting code & software for further information.

Data

Policy information about availability of data

All manuscripts must include a data availability statement. This statement should provide the following information, where applicable:

- Accession codes, unique identifiers, or web links for publicly available datasets
- A list of figures that have associated raw data
- A description of any restrictions on data availability

The source data underlying the graphs and charts presented in the main figures are shown as Supplementary Data. Uncropped blots of major figures are shown in Supplementary Figure 7. All other data supporting the findings of this study are available within the paper and Supplementary Information.

Field-specific reporting

Please select the one below that is the best fit for your research. If you are not sure, read the appropriate sections before making your selection.

Life sciences study design

All studies must disclose on these points even when the disclosure is negative.

Sample size	8 or 10 mice per group were used in the ovarian cancer xenograft models.
Data exclusions	no data were excluded from the analysis.
Replication	All attempts to replicate the experimental findings were successful.
Randomization	The allocation of cells/mice to different treatments was completely random.
Blinding	*Describe whether the investigators were blinded to group allocation during data collection and/or analysis. If blinding was not possible, describe why OR explain why blinding was not relevant to your study.*

Reporting for specific materials, systems and methods

We require information from authors about some types of materials, experimental systems and methods used in many studies. Here, indicate whether each material, system or method listed is relevant to your study. If you are not sure if a list item applies to your research, read the appropriate section before selecting a response.

Materials & experimental systems

n/a	Involved in the study	
☐	☒	Antibodies
☐	☒	Eukaryotic cell lines
☒	☐	Palaeontology
☐	☒	Animals and other organisms
☒	☐	Human research participants
☒	☐	Clinical data

Methods

n/a	Involved in the study	
☒	☐	ChIP-seq
☒	☐	Flow cytometry
☒	☐	MRI-based neuroimaging

Antibodies

Antibodies used	The antibodies used in this study are listed under Methods.
Validation	All antibodies that are commercially available have been tested for species reactivity and application by the manufacturers.

Eukaryotic cell lines

Policy information about cell lines

Cell line source(s)	HEY, CAOV3, SKOV3, and OVCAR-3 cell lines were obtained from American Type Culture Collection. A2780 and A2780 CIS were obtained from European Collection of Cell Cultures. OVCA-433 cell line was provided by Prof. G. Scambia(Catholic University School of Medicine of Rome)
Authentication	The authentication of all cell lines used was performed by short tandem repeat (STR)-profiling.
Mycoplasma contamination	All cell lines were tested negative for mycoplasma contamination.
Commonly misidentified lines (See ICLAC register)	No misidentified cell lines were used in this study.

Animals and other organisms

Policy information about studies involving animals; ARRIVE guidelines recommended for reporting animal research

Laboratory animals	Female nude mice of 6-week old were from Charles River Lab., Milan, Italy
Wild animals	NA
Field-collected samples	This study did not involve samples collected from the field.
Ethics oversight	All the animal experiments were performed in accordance with the Italian Ministry of Health guidelines and protocols after approval by the Animal Welfare Body of Regina Elena Cancer Institute of Rome.

Note that full information on the approval of the study protocol must also be provided in the manuscript.
